# Supplementary material for: How to Improve Healthcare for Patients with Multimorbidity and Polypharmacy in Primary Care: A Pragmatic Cluster-Randomized Clinical Trial of the MULTIPAP Intervention
Source: J Pers Med. 2022 May 6;12(5):752. doi: 10.3390/jpm12050752 (PMC9144280; doi:10.3390/jpm12050752)
Supplement: Supplementary file 1 [file jpm-12-00752-s001.zip › jpm-1629257-supplementary/MULTIPAP GRoup.pdf]

MULTIPAP Group: see Acknowledgements

MULTIPAP GROUP:

Paula Ara-Bardají (Aragonese Institute of Health Sciences (IACS), IIS Aragón, Miguel Servet University Hospital, Zaragoza, Spain), Mercedes Aza-Pascual-Salcedo (EpiChron Research Group, Aragon Health Sciences Institute (IACS), IIS Aragón, REDISSEC ISCIII, Aragon Health Service (SALUD), Zaragoza, Spain), Antonio Gimeno-Miguel (EpiChron Research Group, Aragon Health Sciences Institute (IACS), IIS Aragón, REDISSEC ISCIII, Miguel Servet University Hospital, Zaragoza, Spain), Francisca González Rubio (EpiChron Research Group, Aragon Health Sciences Institute (IACS), IIS Aragón, REDISSEC ISCIII, PCHC Delicias Sur, Aragon Health Service (SALUD), Zaragoza, Spain), Cristina M Lozano Hernández (Research unit. Primary Health Care Management Madrid. Spain), Alessandra Marengoni (Department of Clinical and Experimental Sciences, University of Brescia, Brescia, Italy), Jesús Martín Fernández (Multiprofessional Teaching Unit for Family and Community Care Primary Care PCHC Oeste, Madrid, Spain), Elena Polentinos Castro (Research unit. Primary Health Care Management Madrid. Spain), José María Valderas Martínez (University of Exeter Medical School, Exeter, UK. 22Department), Maria del Pilar Barnestein-Fonseca (CUDECA Foundation, Biomedical Research Institute of Málaga –IBIMA-, Malaga University, Spain), Miguel Domínguez-Santaella (PCHC Victoria, Malaga-Guadalhorce Health District, Andalusian Health Service, Malaga, Spain), Nuria García-Agua-Soler (Department of Pharmacology, Faculty of Medicine, Malaga University, Spain), María Isabel Márquez-Chamizo (PCHC Carranque, Malaga-Guadalhorce Health District, Andalusian Health Service, Málaga, Spain), José María Ruiz-San-Basilio (PCHC Coín, Malaga-Guadalhorce Health District, Andalusian Health Service, Coín, Málaga, Spain), José María Abad-Díez (Department of Health, Social Welfare and Family, Government of Aragon), Marta Alcaraz Borrajo (Subdirectorate General of Pharmacy and Health Products), Gloria Ariza Cardiel (Multiprofessional Teaching Unit for Family and Community Care Primary Care PCHC Oeste, Madrid, Spain), Amaya Azcoaga Lorenzo (PCHC Pintores, Madrid, Spain), José María Abad-Díez (Aragon Health Service (SALUD), Government of Aragon, Zaragoza, Spain), Marta Alcaraz Borrajo (Subdirectorate General of Pharmacy and Health Products), Ana Cristina Bandrés-Liso (EpiChron Research Group, Aragon Health Sciences Institute (IACS), IIS Aragón, REDISSEC ISCIII, Aragon Health Service (SALUD), Zaragoza, Spain), Amaia Calderon-Larrañaga (EpiChron Research Group, Aragon Health Sciences Institute (IACS), IIS Aragón, REDISSEC ISCIII, Zaragoza, Spain, Aging Research Center, Department of Neurobiology, Care Sciences and Society, Karolinska Institute, Stockholm University, Stockholm, Sweden) Mercedes Clerencia-Sierra (EpiChron Research Group, Aragon Health Sciences Institute (IACS), IIS Aragón, REDISSEC ISCIII, Miguel Servet University Hospital, Aragon Health Service (SALUD), Zaragoza, Spain), Javier Marta-Moreno (EpiChron Research Group, Aragon Health Sciences Institute (IACS), IIS Aragón, REDISSEC ISCIII, Miguel Servet University Hospital, Aragon Health Service (SALUD), Zaragoza, Spain), Antonio Poncel-Falcó (EpiChron Research Group, Aragon Health Sciences Institute (IACS), IIS Aragón, REDISSEC ISCIII, Aragon Health Service, Zaragoza, Spain), Ana Isabel González González (Technical Support Unit, Primary Care Management, Madrid Health Service), Virginia Hernández Santiago (Ninewells Hospital & Medical School, Dundee, UK), Angel Mataix SanJuan (Subdirectorate General of Pharmacy and Health Products, Madrid, Spain), Ricardo Rodríguez Barrientos (Research unit. Primary Health Care Management, Madrid, Spain), Mercedes Rumayor Zarzuelo (Public Health Centre Coslada, Área II Subdirectorate of Prevention and Health Promotion, Madrid, Spain), Luis Sánchez Perruca (General System Information Management, Primary Healthcare Management, Madrid Health Service, Spain), Teresa Sanz Cuesta (Research unit. Primary Health Care Management Madrid. Spain), Maria Eugenia Tello Bernabé (PCHC El Naranjo, Madrid, Spain).

Clinical Investigators in Primary Care Health Centres (PCHC):
(Andalucía, Spain): PCHC Alhaurín el Grande (Alhaurin el Grande): Javier Martín Izquierdo, Macarena Toro Sainz. **PCHC Carranque** (Málaga): María José Fernández Jiménez, Esperanza Mora García, José Manuel Navarro Jiménez. **PCHC Ciudad Jardín,** (Málaga): Leovigildo Ginel Mendoza, Luz Pilar de la Mota Ybancos, Jaime Sasporte Genafo. **PCHC Coín,** (Málaga): María José Alcaide Rodríguez, Elena Barceló Garach, Beatriz Caffarena de Arteaga, María Dolores Gallego Parrilla,

Catalina Sánchez Morales. **PCHC Delicias,** (Málaga): Maria del Mar Loubet Chasco, Irene Martínez Ríos, Elena Mateo Delgado. **PCHC La Roca,** (Málaga): Esther Martín Aurioles. **PCHC Limonar,** (Málaga): Sylvia Hazañas Ruiz. **PCHC Palmilla,** (Málaga): María Auxiliadora Nieves Muñoz Escalante. **PCHC Puerta Blanca,** (Málaga): Enrique Leonés Salido, Maria Antonia Máximo Torres, Maria Luisa Moya Rodríguez, María Encarnación Peláez Gálvez, José Manuel Ramírez Torres, Cristóbal Trillo Fernández. **PCHC Tiro Pichón,** (Málaga): Maria Dolores García Martínez Cañavate, Maria del Mar Gil Mellado, Maria Victoria Muñoz Pradilla. **PCHC Vélez Sur** (Vélez Málaga): Maria José Clavijo Peña, José Leiva Fernández, Virginia Castillo Romero. **PCHC Vera,** (Vera, Almería): Rubén Vázquez Alarcón. **PCHC Victoria,** (Málaga): Rafael Ángel Maqueda, Gloria Aycart Valdés, Ana Maria Fernández Vargas, Irene García, Antonia González Rodríguez, Maria Carmen Molina Mendaño, Juana Morales Naranjo, Francisco Serrano Guerra.

(Aragón, Spain): PCHC Alcorisa, (Alcorisa, Teruel): Carmen Sánchez Celaya del Pozo. **PCHC Delicias Norte**, (Zaragoza): José Ignacio Torrente Garrido, Concepción García Aranda, Marina Pinilla Lafuente, Mª Teresa Delgado Marroquín. **PCHC Picarral**, (Zaragoza): Mª José Gracia Molina, Javier Cuartero Bernal, Mª Victoria Asín Martín, Susana García Domínguez. **PCHC Fuentes de Ebro,** (Zaragoza): Carlos Bolea Gorbea. **PCHC Valdefierro,** (Zaragoza): Antonio Luis Oto Negre. **PCHC Actur Norte,** (Zaragoza): Eugenio Galve Royo, Mª Begoña Abadía Taira. **PCHC Alcañiz,** (Teruel): José Fernando Tomás Gutiérrez. **PCHC Sagasta - Ruiseñores,** (Zaragoza): José Porta Quintana, Valentina Martín Miguel, Esther Mateo de las Heras, Carmen Esteban Algora. **PCHC Ejea** (Ejea de los Caballeros, Zaragoza): Maria Teresa Martín Nasarre de Letosa, Elena Gascón del Prim, Noelia Sorinas Delgado, Maria Rosario Sanjuan Cortés. **PCHC Canal Imperial - Venecia,** (Zaragoza): Teodoro Corrales Sánchez. **PCHC Canal Imperial - San José Sur,** (Zaragoza): Eustaquio Dendarieta Lucas. **PCHC Jaca** (Jaca, Huesca): María del Pilar Mínguez Sorio. **PCHC Santo Grial,** (Huesca): Adolfo Cajal Marzal.

(Madrid, Spain): PCHC Mendiguchía Carriche (Leganés): Francisca García De Blas González Eduardo Díaz García, Juan Carlos García Álvarez, Cristina Guisado Pérez, Alberto López García Franco, Maria Elisa Viñuela Benitez. **PCHC El Greco** (Getafe): Ana Ballarín González, Maria Isabel Ferrer Zapata, Esther Gómez Suarez, Fernanda Morales Ortiz, Lourdes Carolina Peláez Laguno, José Luis Quintana Gómez, Enrique Revilla Pascual. **PCHC Cuzco** (Fuenlabrada): M Ángeles Miguel Abanto. **PCHC El Soto** (Móstoles): Blanca Gutiérrez Teira, **PCHC General Ricardos** (Madrid): Francisco Ramón Abellán López, Carlos Casado Álvaro, Paulino Cubero González, Santiago Manuel Machín Hamalainen, Raquel Mateo Fernández, Cesar Sánchez Arce. **PCHC Ibiza** (Madrid): Jorge Olmedo Galindo. **PCHC Las Américas** (Parla): Claudia López Marcos, Soledad Lorenzo Borda, Juan Carlos Moreno Fernández, Belén Muñoz Gómez, Enrique Rodríguez De Mingo. **PCHC Maria Ángeles López** (Leganés): Juan Pedro Calvo Pascual, Margarita Gómez Barroso, Beatriz López Serrano, Maria Paloma Morso Peláez, Julio Sánchez Salvador, Jeannet Dolores Sánchez Yépez, Ana Sosa Alonso. **PCHC Mª Jesús Hereza** (Leganés): Maria del Mar Álvarez Villalba. **PCHC Pavones** (Madrid): Purificación Magán Tapia. **PCHC Pedro Laín Entralgo** (Alcorcón): María Angélica Fajardo Alcántara, Maria Canto De Hoyos Alonso, Maria Aránzazu Murciano Antón. **PCHC Pintores** (Parla): Manuel Antonio Alonso Pérez, Ricardo De Felipe Medina, Amaya Nuria López Laguna, Eva Martínez Cid De Rivera, Iliana Serrano Flores, Maria Jesús Sousa Rodríguez. **PCHC Ramón y Cajal** (Alcorcón): Maria Soledad Núñez Isabel, Jesús Mª Redondo Sánchez, Pedro Sánchez Llanos, Lourdes Visedo Campillo.
