# Supplementary material for: How to Improve Healthcare for Patients with Multimorbidity and Polypharmacy in Primary Care: A Pragmatic Cluster-Randomized Clinical Trial of the MULTIPAP Intervention
Source: J Pers Med. 2022 May 6;12(5):752. doi: 10.3390/jpm12050752 (PMC9144280; doi:10.3390/jpm12050752)
Supplement: Supplementary file 1 [file jpm-12-00752-s001.zip › jpm-1629257-supplementary/Supplementary File S1.pdf]

Prados-Torres et al. Implementation Science (2017) 12:54
DOI 10.1186/s13012-017-0584-x

Implementation Science

STUDY PROTOCOL

Open Access

Effectiveness of an intervention for improving drug prescription in primary care patients with multimorbidity and polypharmacy: study protocol of a cluster randomized clinical trial (Multi-PAP project)

Alexandra Prados-Torres[1,2,3,4*], Isabel del Cura-González[3,5,6], Daniel Prados-Torres[7,8,9], Juan A. López-Rodríguez[3,5,6,10], Francisca Leiva-Fernández[7,8,9], Amaia Calderón-Larrañaga[1,3,4,11], Fernando López-Verde[12], Luis A. Gimeno-Feliu[2,3,13], Esperanza Escortell-Mayor[3,5], Victoria Pico-Soler[3,14], Teresa Sanz-Cuesta[3,5], Mª Josefa Bujalance-Zafra[15], Mariel Morey-Montalvo[3,5], José Ramón Boxó-Cifuentes[16], Beatriz Poblador-Plou[1,3,4], José Manuel Fernández-Arquero[17], Francisca González-Rubio[3,18], María D. Ramiro-González[19], Carlos Coscollar-Santaliestra[3,13], Jesús Martín-Fernández[3,6,20], Mª Pilar Barnestein-Fonseca[8,9], José María Valderas-Martínez[21], Alessandra Marengoni[22], Christiane Muth[23] and Multi-PAP Group

Abstract

Background: Multimorbidity is associated with negative effects both on people's health and on healthcare systems. A key problem linked to multimorbidity is polypharmacy, which in turn is associated with increased risk of partly preventable adverse effects, including mortality. The Ariadne principles describe a model of care based on a thorough assessment of diseases, treatments (and potential interactions), clinical status, context and preferences of patients with multimorbidity, with the aim of prioritizing and sharing realistic treatment goals that guide an individualized management. The aim of this study is to evaluate the effectiveness of a complex intervention that implements the Ariadne principles in a population of young-old patients with multimorbidity and polypharmacy. The intervention seeks to improve the appropriateness of prescribing in primary care (PC), as measured by the medication appropriateness index (MAI) score at 6 and 12 months, as compared with usual care.
(Continued on next page)

* Correspondence: sprados.iacs@aragon.es
[1]Instituto Aragonés de Ciencias de la Salud (IACS), IIS Aragón, Hospital Universitario Miguel Servet, Paseo Isabel La Católica 1-3, 50009 Zaragoza, Spain
[2]Departamento de Microbiología, Medicina Preventiva y Salud Pública, Universidad de Zaragoza, Zaragoza, Spain
Full list of author information is available at the end of the article

© The Author(s). 2017 **Open Access** This article is distributed under the terms of the Creative Commons Attribution 4.0 International License (http://creativecommons.org/licenses/by/4.0/), which permits unrestricted use, distribution, and reproduction in any medium, provided you give appropriate credit to the original author(s) and the source, provide a link to the Creative Commons license, and indicate if changes were made. The Creative Commons Public Domain Dedication waiver (http://creativecommons.org/publicdomain/zero/1.0/) applies to the data made available in this article, unless otherwise stated.

(Continued from previous page)

Methods/Design: Design: pragmatic cluster randomized clinical trial. Unit of randomization: family physician (FP). Unit of analysis: patient. Scope: PC health centres in three autonomous communities: Aragon, Madrid, and Andalusia (Spain). Population: patients aged 65–74 years with multimorbidity (≥3 chronic diseases) and polypharmacy (≥5 drugs prescribed in ≥3 months). Sample size: $n = 400$ (200 per study arm). Intervention: complex intervention based on the implementation of the Ariadne principles with two components: (1) FP training and (2) FP-patient interview. Outcomes: MAI score, health services use, quality of life (Euroqol 5D-5L), pharmacotherapy and adherence to treatment (Morisky-Green, Haynes-Sackett), and clinical and socio-demographic variables. Statistical analysis: primary outcome is the difference in MAI score between T0 and T1 and corresponding 95% confidence interval. Adjustment for confounding factors will be performed by multilevel analysis. All analyses will be carried out in accordance with the intention-to-treat principle.

Discussion: It is essential to provide evidence concerning interventions on PC patients with polypharmacy and multimorbidity, conducted in the context of routine clinical practice, and involving young-old patients with significant potential for preventing negative health outcomes.

Trial registration: Clinicaltrials.gov, NCT02866799

Background

Multimorbidity, the presence of various chronic diseases in the same individual, is the norm among the elderly population and very prevalent in the adult population in most Western European countries [1–3]. In Spain, the latest National Health Survey reports that individuals of over 75 years of age have an average of 3.2 chronic health problems, while the so-called young-old population (65–74 years) has an average of 2.8. Most studies define multimorbidity as the concurrent presence of two or more or three or more chronic diseases; the latter definition is more suitable for the identification of patients with complex health needs [4].

The potential negative health impacts of multimorbidity include reduced quality of life and functional capacity, inadequate use of health services, and increased complications and healthcare costs [5–7]. These effects are partly attributable to the current model of healthcare, which is essentially organized and designed to address diseases individually [8–10].

Although there is emerging evidence to support policy for the management of people with multimorbidity, the effectiveness of interventions is still uncertain [11], as is the case for clinical practice guidelines (CPGs) for patients with comorbidity [12]. The reality is that uncritical application of the recommendations of multiple CPGs for concurrent diseases in the same patient increases the likelihood of polypharmacy, defined by consensus as the simultaneous consumption of five or more drugs [13]. Family physicians (FP) have reported that this is a daily reality in primary care (PC) [14].

Polypharmacy implies an increased risk of medication-related problems such as interactions and adverse drug reactions, underuse of necessary treatments, low adherence, and partly preventable mortality, in particular in older patients [15]. Inappropriate choice of drugs with regard to age is another major problem, for which alternative (safer) approaches have been proposed that are equally or more effective [16].

Multiple approaches have been designed to measure and reduce inappropriate prescribing [17]. Explicit measures assess prescriptions according to predefined criteria related to the properties of the drugs concerned (e.g. Beers [18] and STOPP/START criteria [19]). However, these criteria may fall short in patients with multiple diseases and (interacting) treatments [20]. Therefore, implicit measures are applied to determine the level of appropriateness of prescribing. Based on the clinical judgment of the rater, implicit measures take into account the health status of the individual patient. The implicit method that is most accepted and validated, both internationally and in Spain, is the medication appropriateness index (MAI) [21, 22].

Evidence supporting the effectiveness of interventions to improve outcomes in patients with multimorbidity remains scarce [8, 23]. The Cochrane systematic review by Smith et al. [24] concluded that drug prescribing and adherence tend to improve when interventions target risk factors of multimorbidity and when they focus on key issues or specific functional difficulties affecting patients. Another Cochrane systematic review [25] evaluating the effectiveness of interventions aimed at minimizing the negative effects of polypharmacy concluded that, despite an overall improvement in prescribing by physicians, the effect on other clinical variables such as hospital admissions and quality of life is unclear. For this reason, the authors emphasized the need to incorporate into clinical trial outcome variables of relevance both for clinicians and patients and to evaluate intervention costs.

Various care programmes have been implemented in Spain to address polypharmacy in elderly patients

(>75 years) by means of a systematic medication review in PC, and this strategy is supported by some evidence [26]. However, patients over 75 years of age constitute only part of the population with multimorbidity. Furthermore, these strategies do not meet the requirements of patient-centred care where patient's preferences are taken into consideration. Shared decision making between health professionals and patients is also thought to improve patient's adherence [27].

The sharing of common and realistic treatment goals between physician and patient is essential to tackle multimorbidity in the PC context and is the cornerstone of the Ariadne principles [28]. The implementation of these principles is based on a thorough assessment of the diseases, treatments and potential treatment interactions, global clinical status, and context of the patient by the physician. This allows prioritization of patients' health problems, taking into account their preferences and wishes and ensuring their individualized management and monitoring. Despite numerous studies demonstrating the effectiveness of shared decision making on health outcomes [29–31], the feasibility of implementation and the impact of the Ariadne principles in PC have not been assessed to date, although the potential benefit of implementing such a strategy in routine clinical practice has been recognized [32, 33].

Objectives

Primary

The main objective is to evaluate the effectiveness of a complex PC intervention implementing the Ariadne principles on the improvement of medication appropriateness in the young-old population with polypharmacy and multimorbidity, as measured by the differences of the MAI score at 6 months (T1) to baseline, compared with usual care.

Secondary

(a) To evaluate the effects of the complex intervention on medication appropriateness after 12 months (T2), as well as on the use of health services, patient quality of life, treatment adherence, and medication safety, as compared with usual care.
(b) To study the cost-utility ratio of the intervention as compared with that of usual care.

Method/design

Design

Pragmatic cluster randomized controlled clinical trial with 12 months of follow-up. The unit of randomization is the FP and the unit of analysis is the patient. A cost-utility study will be performed from the perspective of the funder with a time horizon of 1 year.

Scope of study

The scope of the study is the primary care setting of the Spanish national health system.

Study population

The study population includes patients aged 65 to 74 years with multimorbidity and polypharmacy, attending PC health centres in three autonomous communities (ACs) in Spain: Aragon, Madrid, and Andalusia.

FP selection criteria

– Employed in current position for at least 1 year.
– Stable employment situation, with no intention of leaving their position during the course of the study.
– Agree to participate and provide written informed consent.

Patient selection criteria

1. Inclusion criteria:
   – Age 65 to 74 years.
   – Multimorbidity, defined as ≥3 chronic diseases as per O'Halloran [34].
   – Polypharmacy, defined as ≥5 drugs prescribed over at least the 3 months prior to inclusion in the study.
   – At least one visit to the FP in the past year.
   – Agree to participate and provide written informed consent.
2. Exclusion criteria:
   – Institutionalized patients.
   – Life expectancy of less than 12 months, as determined by the FP.
   – Mental and/or physical conditions considered by the FP to prevent fulfilment of study requirements.

Sample size

The sample size was calculated under the hypothesis that the intervention would lead to a difference of at least 2 units in the change in MAI score at 6 months (T1 vs T0) (i.e. a clinically relevant difference) between study groups. Differences in MAI are assumed to be normally distributed in each intervention arm and the variances are assumed to be equal. According to previous studies, the standard deviation of the difference in MAI is 6 units [32–34]. Therefore, the study should be capable of detecting an effect size of 0.3 (2/6). Considering a power of 80% and assuming simple random sampling, the required sample size would be 286 patients (143 patients in the intervention group and 143 in the control group).

The effective sample size in this type of study design depends on the average size of the cluster and the

degree of correlation between individuals in the cluster. Accordingly, it is necessary to adjust the calculated sample size in accordance with the design effect (DE). An average cluster size of 5 patients per FP and an intra-class correlation coefficient of 0.03 [35] produces the following ($DE = 1 + (5 - 1) \times 0.03 = 1.12$) which gives a sample size, corrected for the DE, of 320 patients. Assuming a percentage of losses of 20%, the final sample size required is 400 patients (200 per group). Assuming that each FP will recruit 5 patients, 80 FPs (40 per group) will be required. In each AC, 133 patients will be recruited.

Recruitment

Voluntary participation will be proposed to FPs working in PC health centres in each of the three ACs. Patients will be added to a randomly ordered list of potential participants provided that they fulfil the inclusion criteria. Strategies to improve protocol adherence of FP will be considered (e.g. individual follow-up of protocol's achievements and recognition via e-mail, offer to participate as co-authors in scientific papers, certified training sessions).

Each FP will consecutively select 5 patients from this list. When a patient agrees to participate, the FP will provide them with detailed information about the study, confirm the inclusion/exclusion criteria, and obtain the patient's written informed consent. If they do not agree to participate, data on the patient's age, sex, and reason for nonparticipation will be collected (see Fig. 1).

Randomization

The unit of randomization is the FP and the unit of analysis the patient. Randomization of the FP will be achieved using the treatment assignment module of the Epidat 4.1 program; the proposed intervention will be considered the treatment and usual care considered the control. To ensure an equal number of FPs in each group (intervention and control), the 'balanced groups' option will be selected.

Once all participating FPs have selected their patients and collected the corresponding baseline data, FP randomization will be performed centrally by the Unidad de Apoyo a la Investigación, Gerencia Asistencial de Atención Primaria in Madrid. Subsequently, each FP will receive the information on the study group to which they have been assigned, at which point all patients recruited by him or her will be included in that group.

Intervention

Intervention group

A complex intervention with two phases is conducted:

– First phase: FP training. This will consist of a previously designed training activity, delivered using the massive online open courses (MOOC) format,

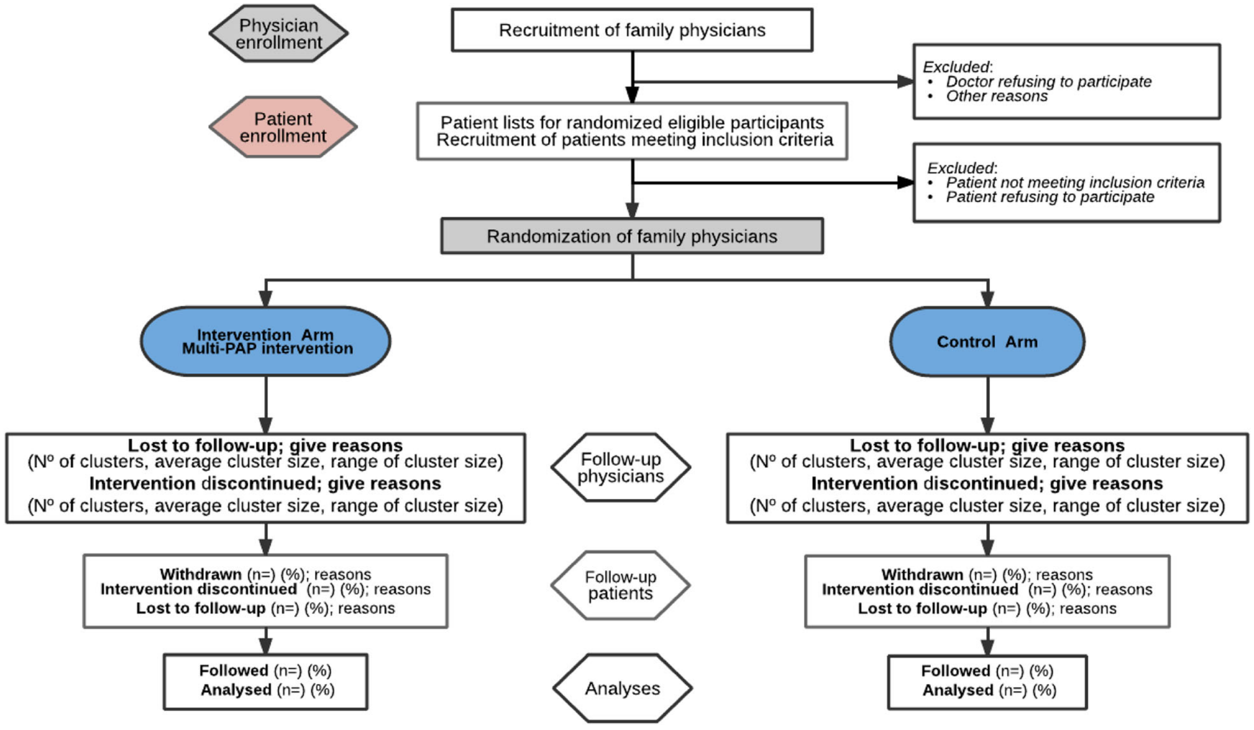

Fig. 1 Flow diagram

including basic concepts relating to multimorbidity, appropriateness of prescribing, treatment adherence, the Ariadne principles, and physician-patient shared decision making.
- Second phase: Physician-patient interview based on the Ariadne principles.

This intervention has been developed in accordance with the recommendations and taxonomy proposed by the Cochrane Effective Practice and Organisation of Care Review Group (EPOC). The intervention is described in detail in Fig. 2, following the approach proposed by Perera et al. [36].

Control group

Patients in the control group will receive usual clinical care based on the provision of advice and information and will undergo examinations as recommended in the CPGs corresponding to each of the patient's chronic diseases.

Variables

FPs will provide their data before the start of the study. Patient data will be collected by the recruiting FP, who will also be responsible for patient follow-up. All information will be recorded in a case report form designed for the study. Each FP will access the form from his/her personal computer via the project website using a personal identification code. Three visits are defined for patient data collection: baseline (T0), 6 months (T1), and 12 months (T2) (see Table 1).

Primary outcome

Appropriateness of prescription will be measured by the medication appropriateness index (MAI). The main outcome variable will be evaluated by an independent FP with training in the MAI. To ensure consistent ratings, an analysis of intra-observer and inter-observer reliability of evaluators will be conducted. Additionally, a FP and a pharmacist will conduct a second appraisal of the inter-observer reliability over a randomly selected 10% of the completed questionnaires. This measure has been proposed in other studies using the MAI [37].

Secondary outcomes

- Use of health services: unplanned and/or avoidable hospitalizations, use of emergency services and PC (FP and nurse).
- Quality of life: measured using the EuroQol 5D-5L questionnaire [38, 39].
- Medication safety: measured as the incidence of adverse drug reactions and potentially hazardous interactions, classified using the taxonomy proposed by Otero-López [40].
- Treatment adherence: measured using the Morisky-Green test [41] and the Haynes-Sackett questionnaire [42].
- Patient perception of shared decision making: measured using a single, multiple choice question, formulated ad hoc.
- Cost-utility: time spent on training FPs, cost of teaching staff, time spent on physician-patient interviews, utilities measured using the EuroQol 5D-5L.

Explanatory and adjustment variables

Patient (first level) variables

- Socio-demographics: age, sex, nationality, AC of residence, marital status, socioeconomic status (monthly salary expressed as multiples of the minimum wage), family composition (number of people living at home), housing indicators, social support (Dukes-UNC-11 questionnaire adapted to Spanish [43]), profession, and social class [44].
- Morbidity: number and description of chronic diseases based on the International Classification of Diseases in PC (ICPC).
- Pharmacotherapeutic treatment plan: number and type of drugs prescribed, active ingredient, and dose of each drug.

FP (second level) variables

- Socio-demographics: age, sex.
- Professional performance: years of professional experience, tutor of residents (yes/no), average workload measured as the average number of daily consultations per FP during the year previous to the start of the study.
- Prior training: in polypharmacy, multimorbidity, and/or shared decision making.

Statistical analysis

All analyses will be carried out in accordance with the intention-to-treat principle, with significance set at $p < 0.05$.

Description of baseline characteristics (qualitative and quantitative variables) of patients and professionals for each arm of the study, with corresponding 95% confidence interval (95% CI). Description of patients that abandon the study, including patient characteristics and reasons for loss during follow-up.

Basal comparison between groups using statistical tests for independent samples (Student's t test or chi-square test). Tests for related samples (ANOVA for repeated

Timeline	Intervention group	Control group
	1	1
Baseline	2	2
Randomization	Randomization of family physicians	
Intervention: family physicians	3 a	
Intervention: patients	4 5	5
Follow-up by nurses	6	6
Outcomes	T1 (6 months)<br>T2 (12 months)	

1	Welcome and training session conducted to the 80 family physicians who participate in the study. Presentation of the project. Training in good practices in research and electronic data capture system (3 hours). This session will be certified and uniformly taught in each participating Autonomous Community.
2	Recruitment of patients. Informed consent. Baseline visit (T0). Before starting the intervention, family physicians will collect all baseline variables: socio-demographics, morbidity, pharmacological treatment plan and QoL.
3	Intervention on family physicians (4 weeks, 5 hours per week). Methodology: active and participatory.<br>- Content (I): multimorbidity and polypharmacy.<br>- Content (II): prescription adequacy and adherence.<br>- Content (III): Ariadne principles and their application to clinical practice.
a	Intervention package and support material. Family physicians will receive written documents and a website link to the MOOC courses (slides, videos, tutorials…).
4	Intervention on patients: structured physician-patient interview. It will comprise a structured review of the treatment plan, inclusion of patient preferences, and a pharmacological treatment plan. Visits 1 and 2.
5	Usual care: clinical follow-up according to available protocols applicable to the patient and the health service standardized portfolio.
6	Follow-up visit by nurses: both groups will receive the usual care plan by their
	corresponding nurses.

Fig. 2 Complex intervention pat plot. *QoL* quality of life, *MOOC* Massive Online Open Courses

measures) will be used to analyse changes within groups and between visits.

Analysis of main effectiveness: between-group difference in T1-T0 MAI score, with corresponding 95% CI. Multilevel analysis will be used to adjust models. Difference in MAI score will be considered the dependent variable; baseline patient (first level) and FP (second level) variables and treatment arm will be considered fixed-effect independent variables; and grouping by FP will be considered a random factor. Missing data pertaining to professionals and/or patients will be addressed by replacing missing values with the most recent available or baseline data.

Analysis of secondary effectiveness (non-confirmatory): between-group difference in means or proportions of T2-T0 MAI score will be determined using the appropriate statistical tests and an explanatory model will be adjusted using the same methodology applied to the main outcome variable.

Estimated quality-adjusted life years (QALYs) gained at the population level, with corresponding 95% CI, as determined using parametric methods and bootstrap techniques. Given the 1-year time horizon, no discount rates will be applied.

Calculation of cost-utility ratio: this is an exploratory objective for which a specific design has not been applied. The cost-utility ratio will be estimated by dividing the total cost by the sum of the potential gains expressed in QALYs. A multivariate sensitivity analysis will be performed in which costs will oscillate within the

Table 1 Visit plan

	T0 (baseline)		T1 (6 months)	T2 (12 months)	Responsible entity
Confirm inclusion/exclusion criteria	X				FP
Written informed consent	X				FP
Socio-demographic variables	X				FP
Morbidity variables and drug treatment plan	X		X	X	FP
Randomization of FPs		X			RU
FP intervention (intervention group)		X			RT
Patient intervention (intervention group)		X	X		FP
Use of health services	X		X	X	RT
Medication adherence	X		X	X	FP
Medication safety			X	X	FP
Quality of life	X		X	X	FP
Costs				X	RT
MAI	X		X	X	EE<br>EC

FP family physician, *RU* research unit, *RT* research team, *EE* external evaluator, *EC* evaluation committee, *MAI* medication appropriateness index

range of uncertainty of a normal distribution. The benefits (QALYs) will also oscillate within the same range.

Discussion

This pragmatic clinical trial will involve the participation of FPs from over 50 PC health centres in different geographic areas of Spain, thus ensuring a high level of external validity, given that the PC model implemented throughout the country is relatively homogeneous.

To address the potential contamination among patients of the same cluster, the FP is considered the unit of randomization and the patient the unit of analysis. Still, there is evidence of contamination when healthcare professionals working in the same teams are randomized. To palliate this problem, the following measures will be introduced. First, during the welcome and training session with all participating FPs, we will avoid sharing too many details regarding the complex intervention [45]. Second, participating FPs will be asked to sign a confidentiality agreement once they are randomly assigned to the intervention or control group. Third, the intervention group will be periodically reminded about the importance of avoiding the exchange of any information with other participating FPs during the intervention. Last, by signing the researcher's commitment, those FP eventually belonging to the control group agree to receive the same training activity once the intervention is over.

Due to the nature of the intervention, it cannot be masked. However, outcome evaluation will be conducted by skilled FPs and pharmacists that are blinded for treatment allocation. The statistician conducting the analysis will neither know to which study arm a given patient has been assigned.

The variability in clinical practice of the different physicians involved and their baseline knowledge on the content covered in the intervention could result in lower than expected differences between groups after completion of the intervention. To address this potential bias, variables related to FPs' prior training in polypharmacy, multimorbidity, and/or shared decision making will be collected and adjusted for in the multilevel model. Furthermore, the MOOC format of the training material will ensure homogenization of the training received by the FPs. Video technology and online courses have shown to be powerful tools to empower both patients and health professionals and have the potential to significantly improve the delivery of care in an increasingly complex healthcare system [46].

FPs who agree to participate in an experimental study are potentially more interested in the subject, just as patients who agree to participate may share certain common features in terms of health-related awareness and motivation, which can lead to bias. Although better results than those obtained including less selective participants may occur, this phenomenon would have a conservative effect (i.e. it would decrease the magnitude of the difference between the two groups). Moreover, participating FPs could modify or improve their prescribing habits just as a response to their awareness of being observed (Hawthorne effect). This may dilute differences between intervention and control groups regarding the appropriateness of prescription.

Although the analysis of the effect of the intervention on quality of life is non-confirmatory and the expected changes are limited over a short period of time, we consider it essential to include this outcome in the study. Firstly, between-group differences may be detected

despite not being highly significant, and secondly, measurement of quality of life changes allows the incorporation of outcome variables reported by patients themselves, and facilitates the calculation of utilities for the cost-utility analysis. This will enable responding to some of the shortcomings and limitations of previous interventions as detected by Patterson et al. [25].

It is essential to provide evidence concerning interventions on PC patients with polypharmacy and multimorbidity, conducted in the context of routine clinical practice, and involving young-old patients with significant potential for preventing negative health outcomes.

Additional files

Additional file 1: Model of the informed consent completed by participants. (DOC 51 kb)

Abbreviations

AC: Autonomous community; CPG: Clinical practice guideline; DE: Design effect; FP: Family physician; ICPC: International classification of diseases in PC; MAI: Medication appropriateness index; MOOC: Massive online open courses; PC: Primary care; QALYs: Quality-adjusted life years

Acknowledgements

Multi-PAP Group:
Mercedes Aza-Pascual-Salcedo (MAP): Dirección de Atención Primaria, Servicio Aragonés de Salud, REDISSEC.
Marta Alcaraz-Borrajo (MAB): Subdirección General de Farmacia y Productos Sanitarios, Servicio Madrileño de Salud.
José Mª Ruiz-San-Basilio (JMR): Centro de Salud de Coín, Distrito Sanitario Málaga/Guadalhorce.
Ángel Mataix-San-Juan (AMSJ): Subdirección General de Farmacia y Productos Sanitarios, Servicio Madrileño de Salud Madrid, REDISSEC.
Ana Mª López-León (ALL): Centro de Salud de Alhaurín el Grande, Distrito Sanitario Málaga/Guadalhorce.
Antonio Poncel-Falcó (APF): Servicio Aragonés de Salud, REDISSEC.
Carmina Mateos-Sancho (CMS): Centro de Salud Ciudad Jardín, Distrito Sanitario Málaga/Guadalhorce.
Antonio Gimeno-Miguel (AGM): IACS, IIS Aragón, Hospital Universitario Miguel Servet, REDISSEC.
Virginia Hernández-Santiago (VHS): Ninewells Hospital & Medical School, Dundee, UK.
Ana Cristina Bandrés-Liso (ABL): Servicio Aragonés de Salud, REDISSEC.
Francisca García-de-Blas (FGB): Centro de Salud Mediguchía Carriche, Servicio Madrileño de Salud, REDISSEC.
Nuria García-Agua (NGA): Departamento de Farmacología, Facultad de Medicina, Universidad de Málaga.
Ricardo Rodríguez-Barrientos (RRB): Unidad de Apoyo Técnico, Gerencia Asistencial de Atención Primaria, Servicio Madrileño de Salud, REDISSEC.
Rubén Vázquez-Alarcón (RVA): Centro de Salud de Vera, AGS Norte de Almería.
Clara Laguna-Berna (CLB): IACS, IIS Aragón, Hospital Universitario Miguel Servet, REDISSEC. JA-CHRODIS.
Maria Isabel Márquez-Chamizo (MMC): Centro de Salud Carranque, Distrito Sanitario Málaga/Guadalhorce.
Javier Marta-Moreno (JMM): Servicio de Neurología, Hospital Universitario Miguel Servet, Servicio Aragonés de Salud, REDISSEC.
Amaya Azcoaga-Lorenzo (AAL): Centro de Salud Pintores, Servicio Madrileño de Salud, REDISSEC.
José María Abad-Díez (JAD): Departamento de Sanidad, Bienestar Social y Familia, Gobierno de Aragón, REDISSEC.
Luis Sánchez-Perruca (LSP): Dirección Sistemas de Información, Gerencia Asistencial de Atención Primaria, Servicio Madrileño de Salud, REDISSEC.
Elena Polentinos-Castro (EPC): Unidad de Apoyo a la Investigación, Gerencia Asistencial de Atención Primaria, Servicio Madrileño de Salud, REDISSEC.
Mercedes Clerencia-Sierra (MCS): Unidad de Valoración Sociosanitaria, Hospital Universitario Miguel Servet, Servicio Aragonés de Salud, REDISSEC.
Gloria Ariza-Cardiel (GAC): Unidad de Apoyo a la Investigación, Gerencia Asistencial de Atención, Servicio Madrileño de Salud, REDISSEC.
Ana Isabel González-González (AIGG): Unidad de Apoyo Técnico, Gerencia Asistencial de Atención Primaria, Servicio Madrileño de Salud, REDISSEC.
Milagros Rico-Blázquez (MRB): Unidad de Apoyo a la Investigación. Gerencia Asistencial de Atención Primaria, Servicio Madrileño de Salud, REDISSEC.
Marisa Rogero-Blanco (MRB): Centro de Salud General Ricardos, Servicio Madrileño de Salud, REDISSEC.
Mª Eugenia Tello-Bernabé (METB): Centro de Salud El Naranjo, Servicio Madrileño de Salud, REDISSEC.
Mar Álvarez-Villalba (MAV): Centro de Salud Mª Jesús Hereza, Servicio Madrileño de Salud, REDISSEC.
Mercedes Rumayor-Zarzuelo (MRZ): Centro de Salud Pública de Coslada, Área II Subdirección de Promoción de la Salud y Prevención, Dirección General de Salud Pública.
Participating primary care health centres (PCHCs) and family physicians in Aragon:
PCHC Alcorisa (Alcorisa): Carmen Sánchez Celaya del Pozo.
PCHC Delicias Norte (Zaragoza): José Ignacio Torrente Garrido, Concepción García Aranda, Marina Pinilla Lafuente, Mª Teresa Delgado Marroquín.
PCHC Picarral (Zaragoza): Mª José Gracia Molina, Javier Cuartero Bernal, Mª Victoria Asín Martín, Susana García Domínguez.
PCHC Fuentes de Ebro (Zaragoza): Carlos Bolea Gorbea.
PCHC Valdefierro (Zaragoza): Antonio Luis Oto Negre.
PCHC Actur Norte (Zaragoza): Eugenio Galve Royo, Mª Begoña Abadía Taira.
PCHC Alcañiz (Alcañiz): José Fernando Tomás Gutiérrez.
PCHC Sagasta - Ruiseñores (Zaragoza): José Porta Quintana, Valentina Martín Miguel, Esther Mateo de las Heras, Carmen Esteban Algora.
PCHC Ejea (Ejea de los Caballeros): Mª Teresa Martín Nasarre de Letosa, Elena Gascón del Prim, Noelia Sorinas Delgado, Mª Rosario Sanjuan Cortés.
PCHC Canal Imperial—Venecia (Zaragoza): Teodoro Corrales Sánchez.
PCHC Canal Imperial—San José Sur (Zaragoza): Eustaquio Dendarieta Lucas.
PCHC Jaca (Jaca): Mª del Pilar Mínguez Sorio.
PCHC Santo Grial (Huesca): Adolfo Cajal Marzal.
Participating PCHCs and family physicians in Madrid:
PHHC Mendiguchía Carriche (Leganés): Eduardo Díaz García, Juan Carlos García Álvarez, Francisca García De Blas González, Cristina Guisado Pérez, Alberto López García Franco, Mª Elisa Viñuela Beneitez.
PCHC El Greco (Getafe): Ana Ballarín González, Mª Isabel Ferrer Zapata, Esther Gómez Suarez, Fernanda Morales Ortiz, Lourdes Carolina Peláez Laguno, José Luis Quintana Gómez, Enrique Revilla Pascual.
PCHC General Ricardos (Madrid): Francisco Ramón Abellán López, Carlos Casado Álvaro, Paulino Cubero González, Santiago Manuel Machín Hamalainen, Raquel Mateo Fernández, Mª Eloisa Rogero Blanco, Cesar Sánchez Arce.
PHHC Gregorio Marañón (Alcorcón): Elisa Ceresuela Wiesman.
PCHC Ibiza (Madrid): Jorge Olmedo Galindo.
PCHC Las Américas (Parla): Claudia López Marcos, Soledad Lorenzo Borda, Juan Carlos Moreno Fernández, Belén Muñoz Gómez, Enrique Rodríguez De Mingo.
PCHC Mª Ángeles López (Leganés): Juan Pedro Calvo Pascual, Margarita Gómez Barroso, Beatriz López Serrano, Mª Paloma Morso Peláez, Fernando Perales González, Julio Sánchez Salvador, Jeannet Dolores Sánchez Yépez, Ana Sosa Alonso.
PCHC Mª Jesús Hereza (Leganés): Mª del Mar Álvarez Villalba.
PCHC Pavones (Madrid): Purificación Magán Tapia.
PCHC Pedro Laín Entralgo (Alcorcón): Mª Angelica Fajardo Alcántara, Mª Canto De Hoyos Alonso, Rosario Iglesias González, Mª Aránzazu Murciano Antón.
PCHC Pintores (Parla): Manuel Antonio Alonso Pérez, Amaya Azcoaga Lorenzo, Ricardo De Felipe Medina, Amaya Nuria López Laguna, Eva Martínez Cid De Rivera, Iliana Serrano Flores, Mª Jesús Sousa Rodríguez.
PCHC Ramón y Cajal (Alcorcón): Mª Soledad Núñez Isabel, Jesús Mª Redondo Sánchez, Pedro Sánchez Llanos, Lourdes Visedo Campillo.
Participating PCHCs and family physicians in Andalusia:
PCHC Alhaurín el Grande: Javier Martín Izquierdo, Macarena Toro Sainz.
PCHC Carranque: Mª José Fernández Jiménez, Esperanza Mora García, José Manuel Navarro Jiménez.
PCHC Ciudad Jardín: Deborah Gil Gómez, Leovigildo Ginel Mendoza, Luz Pilar de la Mota Ybancos, Jaime Sasporte Genafo.
PCHC Coín: Mª José Alcaide Rodríguez, Elena Barceló Garach, Beatriz Caffarena de Arteaga, Mª Dolores Gallego Parrilla, Catalina Sánchez Morales.

PCHC Delicias: Mª del Mar Loubet Chasco, Irene Martínez Ríos, Elena Mateo Delgado.
PCHC La Roca: Esther Martín Aurioles.
PCHC Limonar: Sylvia Hazañas Ruiz.
PCHC Palmilla: Nieves Muñoz Escalante.
PCHC Puerta Blanca: Enrique Leonés Salido, Mª Antonia Máximo Torres, Mª Luisa Moya Rodríguez, Encarnación Peláez Gálvez, José Manuel Ramírez Torres, Cristóbal Trillo Fernández.
PCHC Tiro Pichón: Mª Dolores García Martínez Cañavate, Mª del Mar Gil Mellado, Mª Victoria Muñoz Pradilla.
PCHC Vélez Sur: Mª José Clavijo Peña, José Leiva Fernández, Virginia Castillo Romero.
PCHC Victoria: Rafael Ángel Maqueda, Gloria Aycart Valdés, Miguel Domínguez Santaella, Ana Mª Fernández Vargas, Irene García García, Antonia González Rodríguez, Mª Carmen Molina Mendaño, Juana Morales Naranjo, Catalina Moreno Torres, Francisco Serrano Guerra.

Funding
This study was funded by the Fondo de Investigaciones Sanitarias ISCIII (Grant Numbers PI15/00276, PI15/00572, PI15/00996), REDISSEC (Project Numbers RD12/0001/0012, RD16/0001/0005), and the European Regional Development Fund ("A way to build Europe"). Funders had no role in study design or in the decision to submit the report for publication.

Availability of data and materials
Family physicians will have access to personal data of their own patients. Anonymised final trial dataset will be accessed only by members of the research team. Investigators will offer the possibility of communicating trial results to participants and healthcare professionals under request. Anonymised trial results will be updated in Clinicaltrials.gov for the general public.

Authors' contributions
APT, IDC, and DPT conceived the study and participated in its design and coordination. JALR, EEM, TSC, MMM, JMF, MDRG, MAB, ACL, BPP, LGF, FGR, VPS, CCS, CLB, AGM, FLF, FLV, JBZ, JRB, JMFA, VHS, JMV, CM, AM, AAL, RRB, GAC, EPC, MRB, AIGG, AMSJ; MRB, MAV, MRZ, METB, FGB, MAP, MCS, APF, JMM, JAD, ABL, CMS, MMC, NGA, JMR, ALL, PBF, and RVA participated in different phases of the protocol study design. IDC, JALR, EEM, TSC, APT, ACL, BPP, DPT, and FLF collaborated in the writing of the manuscript. IDC, JALR, APT, ACL, DPT, and FLF wrote the final manuscript. All authors from the Multi-PAP group have read and approved the final manuscript.

Competing interests
The authors declare that they have no competing interests.

Consent for publication
Not applicable.

Ethics approval and consent to participate
The trial is designed in accordance with the basic ethical principles of autonomy, beneficence, justice, and non-maleficence and will be conducted in accordance with the rules of Good Clinical Practice outlined in the most recent Declaration of Helsinki [47] and the Oviedo Convention (1997). Written informed consent of patients will be required. Data confidentiality and anonymity will be ensured, according to the provisions of Spanish Law 15/1999, both during the implementation phase of the project and in any resulting presentations or publications.
The project has been favourably evaluated by the Central Committee of Primary Care Research of the Community of Madrid, the Commission for Health Research of the Aragon Institute for Health Research (IIS Aragon), and the Commission for Health Research of the Bio-Sanitary Research Institute in Malaga (IBIMA). The trial protocol was approved by the Clinical Research Ethics Committee of Aragon (CEICA) on September 30, 2015, and by the Research Ethics Committee of the Province of Malaga on September 25, 2015. A model of the informed consent completed by the participants of this trial is provided as an Additional file 1.
Any important protocol modification will be communicated to the CEICA, updated in Clinicaltrials.gov and reflected in the explanatory memory sent to the Fondo de Investigaciones Sanitarias ISCIII.

Publisher's Note
Springer Nature remains neutral with regard to jurisdictional claims in published maps and institutional affiliations.

Author details
[1]Instituto Aragonés de Ciencias de la Salud (IACS), IIS Aragón, Hospital Universitario Miguel Servet, Paseo Isabel La Católica 1-3, 50009 Zaragoza, Spain. [2]Departamento de Microbiología, Medicina Preventiva y Salud Pública, Universidad de Zaragoza, Zaragoza, Spain. [3]Red de Investigación en Servicios de Salud en Enfermedades Crónicas (REDISSEC) ISCIII, Madrid, Spain. [4]Joint Action on Chronic Diseases (JA-CHRODIS) European Comission, Brussels, Belgium. [5]Unidad de Apoyo a la Investigación, Gerencia Asistencial de Atención Primaria, Servicio Madrileño de Salud, Madrid, Spain. [6]Área Medicina Preventiva y Salud Pública. Facultad de Ciencias de la Salud, Universidad Rey Juan Carlos, Madrid, Spain. [7]Unidad Docente Multiprofesional de Atención Familiar y Comunitaria de Distrito Málaga/ Guadalhorce, Málaga, Spain. [8]Instituto de Investigación Biomédica de Málaga (IBIMA), Distrito Sanitario Málaga/Guadalhorce, Málaga, Spain. [9]Universidad de Málaga, Málaga, Spain. [10]Centro de Salud General Ricardos, Madrid, Spain. [11]Karolinska Institutet, Aging Research Center, Stockholm, Sweden. [12]Centro de Salud las Delicias, Distrito Sanitario Málaga/Guadalhorce, Málaga, Spain. [13]Centro de Salud San Pablo, Zaragoza, Spain. [14]Centro de Salud Torrero-La Paz, Zaragoza, Spain. [15]Centro de Salud la Victoria, Distrito Sanitario Málaga/ Guadalhorce, Málaga, Spain. [16]Centro de Salud Puerta Blanca, Distrito Sanitario Málaga/Guadalhorce, Málaga, Spain. [17]Servicio de Farmacia de Atención Primaria, Distrito Sanitario Málaga/Guadalhorce, Málaga, Spain. [18]Centro de Salud Delicias Sur, Zaragoza, Spain. [19]Servicio de Medicina Preventiva, Hospital Universitario Gregorio Marañón, Madrid, Spain. [20]Centro de Salud de Villamanta, Madrid, Spain. [21]University of Exeter Medical School, Exeter, UK. [22]Department of Clinical and Experimental Sciences, University of Brescia, Brescia, Italy. [23]Institute of General Practice, Johann Wolfgang Goethe University, Frankfurt, Germany.

Received: 22 March 2017 Accepted: 18 April 2017
Published online: 27 April 2017

References
1. Valderas JM, Starfield B, Sibbald B, Salisbury C, Roland M. Defining comorbidity: implications for understanding health and health services. Ann Fam Med. 2009;7:357–63.
2. Prados-Torres A, Poblador-Plou B, Calderon-Larrañaga A, Gimeno-Feliu LA, Gonzalez-Rubio F, Poncel-Falco A, et al. Multimorbidity patterns in primary care: interactions among chronic diseases using factor analysis. PLoS One. 2012;7:e32190.
3. Violan C, Foguet-Boreu Q, Flores-Mateo G, Salisbury C, Blom J, Freitag M, et al. Prevalence, determinants and patterns of multimorbidity in primary care: a systematic review of observational studies. PLoS One. 2014;9:e102149.
4. Harrison C, Britt H, Miller G, Henderson J. Examining different measures of multimorbidity, using a large prospective cross-sectional study in Australian general practice. BMJ Open. 2014;4:e004694.
5. Fortin M, Bravo G, Hudon C, Lapointe L, Almirall J, Dubois M-FF, et al. Relationship between multimorbidity and health-related quality of life of patients in primary care. Qual Life Res. 2006;15:83–91.
6. Gijsen R, Hoeymans N, Schellevis FG, Ruwaard D, Satariano WA, van den Bos GAMM. Causes and consequences of comorbidity: a review. J Clin Epidemiol. 2001;54:661–74.
7. Wolff JL, Starfield B, Anderson G. Prevalence, expenditures, and complications of multiple chronic conditions in the elderly. Arch Intern Med. 2002;162:2269–76.
8. Starfield B. New paradigms for quality in primary care. Br J Gen Pract. 2001;51:303–9.
9. Starfield B, Lemke KW, Bernhardt T, Foldes SS, Forrest CB, Weiner JP. Comorbidity: implications for the importance of primary care in "case" management. Ann Fam Med. 2003;1:8–14.
10. Valderas JM. Multimorbidity, not a health condition or complexity by another name. Eur J Gen Pract. 2015;21:213–4.
11. Mangin D, Heath I, Jamoulle M. Beyond diagnosis: rising to the multimorbidity challenge. BMJ. 2012;344:e3526.
12. Tinetti ME, Bogardus STSTJ, Agostini JV. Potential pitfalls of disease-specific guidelines for patients with multiple conditions. N Engl J Med. 2004;351:2870–4.

13. Blasco Patiño F, de Letona JML, Villares P, Jiménez A, et al. El paciente anciano polimedicado : efectos sobre su salud y sobre el sistema sanitario. Inf Ter del Sist Nac Salud. 2005;29:152–62.
14. Luijks HD, Loeffen MJW, Lagro-Janssen AL, van Weel C, Lucassen PL, Schermer TR, et al. GPs' considerations in multimorbidity management: a qualitative study. Br J Gen Pract. 2012;62:e503–10.
15. Jyrkkä J, Enlund H, Korhonen MJ, Sulkava R, Hartikainen S. Polypharmacy status as an indicator of mortality in an elderly population. Drugs Aging. 2009;26:1039–48.
16. Galván-Banqueri M, González-Méndez AI, Alfaro-Lara ER, Nieto-Martín MD, Pérez-Guerrero C, Santos-Ramos B. Evaluación de la adecuación del tratamiento farmacológico en pacientes pluripatológicos. Aten Primaria. 2013;45:235–43.
17. Ramírez FB. Methods for measuring the suitability of pharmacological treatment in the elderly with multiple conditions and on multiple drugs. Aten Primaria. 2013;45:19–20.
18. Beers MH. Explicit criteria for determining potentially inappropriate medication use by the elderly. An update Arch Intern Med. 1997;157:1531–6.
19. Leendertse AJ, Egberts ACG, Stoker LJ, van den Bemt PML. Frequency of and risk factors for preventable medication-related hospital admissions in the Netherlands. Arch Intern Med. 2008;168:1890–6.
20. Spinewine A, Schmader KE, Barber N, Hughes C, Lapane KL, Swine C, et al. Appropriate prescribing in elderly people: how well can it be measured and optimised? Lancet. 2007;370:173–84.
21. Hanlon JT, Schmader KE, Samsa GP, Weinberger M, Uttech KM, Lewis IK, et al. A method for assessing drug therapy appropriateness. J Clin Epidemiol. 1992;45:1045–51.
22. Gavilán Moral E, Villafaina Barroso A, Aranguez Ruiz A, Sánchez Robles GA, Suliman Criado S, Jiménez de Gracia L. Índice de adecuación de los medicamentos - Versión española modificada manual de usuario. Plasencia: Laboratorio de Prácticas Innovadoras en Polimedicación y Salud. 2012.
23. Fortin M, Dionne J, Pinho G, Gignac J, Almirall J, Lapointe L, et al. Randomized controlled trials : do they have external validity for patients with multiple comorbidities ? Ann Fam Med. 2006;4:104–8.
24. Smith SM, Soubhi H, Fortin M, Hudon C, O'Dowd T. Interventions for improving outcomes in patients with multimorbidity in primary care and community settings. Cochrane Database Syst Rev. 2012;4:CD006560.
25. Patterson SM, Cadogan CA, Kerse N, Cardwell CR, Bradley MC, Ryan C, et al. Interventions to improve the appropriate use of polypharmacy for older people. Cochrane Database Syst Rev. 2014;10:CD008165.
26. Pham CB, Dickman RL. Minimizing adverse drug events in older patients. Am Fam Physician. 2007;76:1837–44.
27. Stevenson FA, Cox K, Britten N, Dundar Y. A systematic review of the research on communication between patients and health care professionals about medicines: the consequences for concordance. Heal Expect. 2004;7:235–45.
28. Muth C, van den Akker M, Blom JW, Mallen CD, Rochon J, Schellevis FG, et al. The Ariadne principles: how to handle multimorbidity in primary care consultations. BMC Med. 2014;12:223.
29. England NHS. Tools for shared decision making. 2014.
30. Elwyn G, Laitner S, Coulter A, Walker E, Watson P, Thomson R. Implementing shared decision making in the NHS. BMJ. 2010;341:c5146.
31. Da Silva D. Helping people share decision making. Heal. Found. London: Health Foundation; 2012.
32. Bower P. Better management of multimorbidity: a critical look at the "Ariadne principles". BMC Med. 2014;12:222.
33. National Institute for Health and Care Excellence. Multimorbidity: clinical assessment and management. 2016.
34. O'Halloran J, Miller GC, Britt H. Defining chronic conditions for primary care with ICPC-2. Fam Pract. 2004;21:381–6.
35. Adams G, Gulliford MC, Ukoumunne OC, Eldridge S, Chinn S, Campbell MJ. Patterns of intra-cluster correlation from primary care research to inform study design and analysis. J Clin Epidemiol. 2004;57:785–94.
36. Perera R, Heneghan C, Yudkin P. Graphical method for depicting randomised trials of complex interventions. BMJ. 2007;334:127–9.
37. Samsa GP, Hanlon JT, Schmader KE, Weinberger M, Clipp EC, Uttech KM, et al. A summated score for the medication appropriateness index: development and assessment of clinimetric properties including content validity. J Clin Epidemiol. 1994;47:891–6.
38. Herdman M, Gudex C, Lloyd A, Janssen M, Kind P, Parkin D, et al. Development and preliminary testing of the new five-level version of EQ-5D (EQ-5D-5 L). Qual Life Res. 2011;20:1727–36.
39. van Hout B, Janssen MFF, Feng Y-SS, Kohlmann T, Busschbach J, Golicki D, et al. Interim scoring for the EQ-5D-5L: mapping the EQ-5D-5L to EQ-5D-3L value sets. Value Health. 2012;15:708–15.
40. Otero López MJ, Codina Jané C, Tamés Alonso MJ, Pérez EM. Medication errors: standarizing the terminology and taxomany. Ruiz Jarabo 2000 grand results. Farm Hosp. 2003;27:137–49.
41. Morisky DE, Green LW, Levine DM. Concurrent and predictive validity of a self-reported measure of medication adherence. Med Care. 1986;24:67–74.
42. Haynes RB, Sackett DL, Taylor DW. How to detect and manage low patient compliance in chronic illness. Geriatrics. 1980;35:91–3. 96–7.
43. Bellón Saameño JA, Delgado Sánchez A, Luna del Castillo JD, Lardelli Claret P. Validity and reliability of the Duke-UNC-11 questionnaire of functional social support. Aten Primaria. 1996;18:153–6. 158–63.
44. Domingo-Salvany A, Bacigalupe A, Carrasco JM, Espelt A, Ferrando J, Borrell C. Propuestas de clase social neoweberiana y neomarxista a partir de la Clasificación Nacional de Ocupaciones 2011. Gac Sanit. 2013;27:263–72.
45. Muth C, Harder S, Uhlmann L, Rochon J, Fullerton B, Güthlin C, et al. Pilot study to test the feasibility of a trial design and complex intervention on PRI oritising MU ltimedication in M ultimorbidity in general practices (PRIMUM pilot). BMJ Open. 2016;6:e011613.
46. Volandes AE, Kennedy WJ, Davis AD, Gillick MR, Paasche-Orlow MK. The new tools: what 21st century education can teach us. Healthc. 2013;1:79–81.
47. World Health Organization. Declaration of Helsinki World Medical Association Declaration of Helsinki: ethical principles for medical research involving human subjects. J Am Med Assoc. 2013;310:2191–4.

Submit your next manuscript to BioMed Central and we will help you at every step:

- We accept pre-submission inquiries
- Our selector tool helps you to find the most relevant journal
- We provide round the clock customer support
- Convenient online submission
- Thorough peer review
- Inclusion in PubMed and all major indexing services
- Maximum visibility for your research

Submit your manuscript at www.biomedcentral.com/submit

BioMed Central
