# Supplementary material for: How to Improve Healthcare for Patients with Multimorbidity and Polypharmacy in Primary Care: A Pragmatic Cluster-Randomized Clinical Trial of the MULTIPAP Intervention
Source: J Pers Med. 2022 May 6;12(5):752. doi: 10.3390/jpm12050752 (PMC9144280; doi:10.3390/jpm12050752)
Supplement: Supplementary file 1 [file jpm-12-00752-s001.zip › jpm-1629257-supplementary/Supplementary Table S2.pdf]

Supplementary Table S2.

TIDieR
Template for Intervention Description and Replication

The TIDieR (Template for Intervention Description and Replication) Checklist*:

Information to include when describing an intervention and the location of the information

Item number	Item<br>MULTIPAP COMPLEX INTERVENTION	Where located **<br>Primary paper (page or appendix number)	Other † (details)
	**BRIEF NAME**		
**1.**	Provide the name or a phrase that describes the intervention. **MULTIPAP**	______________	________________
	**WHY**		
**2.**	Describe any rationale, theory, or goal of the elements essential to the intervention.<br>Multimorbidity and polypharmacy imply a higher risk of drug-related problems, such as drug interactions and adverse reactions, under-use of necessary treatments, low adherence and partially preventable mortality, particularly in elderly patients.<br><br>MULTIPAP complex intervention is based on the implementation of the Ariadne principles. These principles form a basis for establishing realistic treatment objectives, agreed upon by both the doctor and patient, after an initial phase of identification and prioritization of the patient's health problems and preferences.<br>The main educational objective is to review international recommendations for the management of MM and polypharmacy in primary care, minimizing to the extent possible the risks to patient safety and, especially, those associated with the drug combinations usually administered in this population, as well as therapeutic cascade	______________	_______________
	**WHAT**		

**3.**	Materials: Describe any physical or informational materials used in the intervention, including those provided to participants or used in intervention delivery or in training of intervention providers. Provide information on where the materials can be accessed (e.g. online appendix, URL).	______________	______________
**4.**	Procedures: Describe each of the procedures, activities, and/or processes used in the intervention, including any enabling or support activities.	______________	______________

MULTIPAP complex intervention is based on the implementation of the Ariadne principles. The MULTIPAP intervention has been developed in accordance with the recommendations and taxonomy proposed by the Cochrane Effective Practice and Organisation of Care Review Group (EPOC).

The two components of the MULTIPAP intervention are FP training and an enhanced FP–patient interview based on the international consensus on best care.

The FP training was based on the completion of eMULTIPAP, a structured “ad hoc” designed course based on constructivism and problem-based learning. The eMULTIPAP course has been assessed according to the Kirkpatrick model and has shown knowledge improvement and high applicability of learning with more motivation to consider multimorbidity in the clinical practice, addressing Lewis' proposed curriculum for multimorbidity[27] and including -multimorbidity, polypharmacy- appropriateness of prescribing, treatment adherence, the Ariadne principles, and physician–patient shared decision making basic concepts. The type 4 logic model for this intervention can be found elsewhere[28]

The course consists of 4 modules and lasts 4 weeks. Each week, students are provided with access to the training content corresponding to a module, which then remains accessible for the remainder of the course.

The eMULTIPAP course contents were developed in videos, presentations, recommended readings, evaluative activities, and forum, and offers students training in the detection of polypharmacy-associated problems (e.g. adverse reactions, interactions, lack of treatment adherence), deprescribing as a strategy to manage polypharmacy, and the conceptual framework of the Ariadne principles.

In addition, students are provided with tools to put these principles into practice, with an emphasis on the doctor-patient relationship, patient adherence to treatment, and the importance of taking patient opinions and preferences into account through the use of a model focused on patient-centred care.

WEEK 1 Introduction to Multimorbidity and polypharmacy

WEEK 2 Polypharmacy. Inappropriate prescribing, therapeutic reconciliation, and treatment adherence

WEEK 3 The Ariadne Principles and its application in clinical practice

WEEK 4 Practical tools to apply The Ariadne Principles in clinical practice

FPs in the control group continued to provide usual care. Usual care is usually provided based on recommendations from the clinical practice guidelines and protocols corresponding to each separate patient chronic disease.

WHO PROVIDED

5. For each category of intervention provider (e.g. psychologist, nursing assistant), describe their expertise, background and any specific training given. ______________ ______________

a) Family physicians training: eMULTIPAP. The content was designed by researchers working on the MULTIPAP project. The students were tutored in each module by at least two expert researchers from each module. The total number of doctors enrolled the eMULTIPAP course was 117: 59 in the first edition, corresponding to the intervention group of the MULTIPAP study; and 58 in the second edition, corresponding to the control group.

b) Patient interview: Family physicians who have received eMULTIPAP course.

.

HOW

6.	a) The course has been designed for presentation in an electronic format, using the online platform MOODLE (Modular Object Oriented Dynamic Learning Environment).<br>b) Structured physician-patient interview, comprising a review of the treatment plan, inclusion of patient preferences, and a pharmacological treatment plan. Follow-up visit by nurses according to usual care and protocols.	______________	______________
	**WHERE**		
7.	Describe the type(s) of location(s) where the intervention occurred, including any necessary infrastructure or relevant features.<br>Thirty-eight primary care centres (clinical practice) in three Spanish regions (Aragon, Madrid and Andalusia).	______________	______________
	**WHEN and HOW MUCH**		
8.	Describe the number of times the intervention was delivered and over what period of time including the number of sessions, their schedule, and their duration, intensity or dose.<br>The course consists of 4 modules and lasts 4 weeks. Each weekly module consists of the following learning materials: 10–15-minute videos (video transcripts are also provided), slide presentations, recommended reading, and “learn more” links. The evaluative activities in which the students participated were Simulated Multimorbidity Cases and questionnaires consisting of multiple-choice questions. The module also included supervised interactive forums, both specific and general.<br>In the intervention group, the family physicians were required to visit the patient after the end of the intervention. In the control group, they continued their usual practice.	______________	______________
	**TAILORING**		
9.	If the intervention was planned to be personalised, titrated or adapted, then describe what, why, when, and how.<br>The module also included supervised interactive forums, both specific and general.	______________	______________
	**MODIFICATIONS**		

**10.‡**	If the intervention was modified during the course of the study, describe the changes (what, why, when, and how).<br>No modifications		
	**HOW WELL**		
**11.**	Planned: If intervention adherence or fidelity was assessed, describe how and by whom, and if any strategies were used to maintain or improve fidelity, describe them.		
**12.‡**	Actual: If intervention adherence or fidelity was assessed, describe the extent to which the intervention was delivered as planned.<br><br>117 Family physicians were enrolled to the eMULTIPAP course. Of them, 107 (91.4%) completed the training activity, 59 (100%) and 48 (82.7%) in the first and second editions, respectively. The remaining participants failed to complete some of the course tasks<br>The training impact of the eMULTIPAP course was evaluated using the Kirkpatrick model<br><u>The first level of evaluation (Reaction):</u> a cross-sectional descriptive study was conducted to evaluate student participation in the activity (i.e., the percentage of students that completed training after performing all proposed tasks) and their level of satisfaction.<br><u>The second level of evaluation (Learning):</u> a before-and-after study was conducted to evaluate knowledge gain using a questionnaire consisting of 15 multiple-choice test questions, each with a single valid answer.<br><u>The third level of evaluation (Behaviour):</u> analysed behavioural changes in participating professionals as self-reported at least 3 months after completing the course. This was achieved by performing a descriptive analysis of the GPs who had completed the eMULTIPAP course. A self-administered questionnaire was completed by all participants on the same online platform as used to deliver the training activity<br><u>The fourth level of evaluation (Results):</u> To measure changes in clinical practice resulting from the training and consequent benefits for patients, the following parameters were assessed as part of the MULTIPAP clinical trial: a) inappropriate prescriptions, assessed using the MAI (Medication Appropriateness Index) tool; b) use of		

	services by patients (i.e., unscheduled and/or avoidable hospitalizations, visits to the doctor and/or nurse in the emergency department and/or in primary care); c) quality of life as perceived by the patients (Euroqol 5D-5L questionnaire); and d) patient adherence to treatment.	

** **Authors** - use N/A if an item is not applicable for the intervention being described. **Reviewers** – use '?' if information about the element is not reported/not sufficiently reported.

† If the information is not provided in the primary paper, give details of where this information is available. This may include locations such as a published protocol or other published papers (provide citation details) or a website (provide the URL).

‡ If completing the TIDieR checklist for a protocol, these items are not relevant to the protocol and cannot be described until the study is complete.

* We strongly recommend using this checklist in conjunction with the TIDieR guide (see *BMJ* 2014;348:g1687) which contains an explanation and elaboration for each item.

* The focus of TIDieR is on reporting details of the intervention elements (and where relevant, comparison elements) of a study. Other elements and methodological features of studies are covered by other reporting statements and checklists and have not been duplicated as part of the TIDieR checklist. When a **randomised trial** is being reported, the TIDieR checklist should be used in conjunction with the CONSORT statement (see www.consort-statement.org) as an extension of **Item 5 of the CONSORT 2010 Statement.** When a **clinical trial protocol** is being reported, the TIDieR checklist should be used in conjunction with the SPIRIT statement as an extension of **Item 11 of the SPIRIT 2013 Statement** (see www.spirit-statement.org). For alternate study designs, TIDieR can be used in conjunction with the appropriate checklist for that study design (see www.equator-network.org).
