# Supplementary material for: How to Improve Healthcare for Patients with Multimorbidity and Polypharmacy in Primary Care: A Pragmatic Cluster-Randomized Clinical Trial of the MULTIPAP Intervention
Source: J Pers Med. 2022 May 6;12(5):752. doi: 10.3390/jpm12050752 (PMC9144280; doi:10.3390/jpm12050752)
Supplement: Supplementary file 1 [file jpm-12-00752-s001.zip › jpm-1629257-supplementary/Supplementary Table S3.pdf]

Supplementary Table S3. Comparison between patients excluded and enrolled.

	**Excluded**	**Enrolled**	**P value**
	n=42	n=593	
**Sex**			
Male	16 (38.1%)	262 (44.2%)	0.44
Female	26 (61.9%)	331 (55.8%)	
**Age, mean (SD)**	70.7 (2.9)	69.7 (2.7)	***0.02***
**Autonomous Community**			
Andalusia	14 (33.3%)	199 (33.6%)	
Aragon	10 (23.81%)	138 (23.3%)	0.997
Madrid	18 (42.86%)	256 (43.2%)	
