# Supplementary material for: How to Improve Healthcare for Patients with Multimorbidity and Polypharmacy in Primary Care: A Pragmatic Cluster-Randomized Clinical Trial of the MULTIPAP Intervention
Source: J Pers Med. 2022 May 6;12(5):752. doi: 10.3390/jpm12050752 (PMC9144280; doi:10.3390/jpm12050752)
Supplement: Supplementary file 1 [file jpm-12-00752-s001.zip › jpm-1629257-supplementary/Supplementary Table S4.pdf]

Supplementary Table S4. Sensitivity analyses of the primary outcome at 6-month follow-up.

**Difference in MAI T1-T0 mean (SD)**	**Control group** N=295	**Intervention group** N=298	**Adjusted difference in means (95% CI); p value**
Per-protocol analysis	1.06 (6.75); n=283	3.55 (10.4); n=287	-2.48(-3.92; -1.04) p=0.001
Intention-to-treat analysis LOCF	1.03 (6.65); n=292	3.43 (10.24); n=297	-2.39(-3.80; -1.00); p=0.0008
Intention-to-treat analysis multiple imputation	1.08 (0.41); n=295	3.43 (0.84); n=298	-2.42(-4.27; -0.59); p=0.009
