# Supplementary material for: How to Improve Healthcare for Patients with Multimorbidity and Polypharmacy in Primary Care: A Pragmatic Cluster-Randomized Clinical Trial of the MULTIPAP Intervention
Source: J Pers Med. 2022 May 6;12(5):752. doi: 10.3390/jpm12050752 (PMC9144280; doi:10.3390/jpm12050752)
Supplement: Supplementary file 1 [file jpm-12-00752-s001.zip › jpm-1629257-supplementary/Supplementary Table S5.pdf]

Supplementary Table S5. Sub-group analyses of the primary outcome at 6-month follow-up (intention-to-treat analysis).

	**Control group**		**Intervention group**				
	Unadjusted mean difference in MAI (SD)	N	Unadjusted mean difference in MAI (SD)	N	Unadjusted difference in means	Adjusted difference in means*	Interaction term
**Age**							
Patients by median age	1.03 (6.65)	292	3.43 (10.24)	297	-2.4 (-3.8; -1)	-1.85 (-3.38; -0.32)	-
<70 years	1.36 (6.63)	128	2.87 (10.86)	134	-1.51 (-3.71; 0.7)	-0.78 (-2.85; 1.29)	
≥70 years	0.77 (6.68)	164	3.89 (9.72)	163	-3.12 (-4.93; -1.3)	-2.64 (-4.39; -0.89)	-1.99 (-4.32;0.34)
**Number of long-term conditions**							
Three	1.62 (7.83)	29	2.59 (12.54)	41	-0.96 (-6.22; 4.29)	0.2 (-5.07; 5.47)	
Four or more	0.96 (6.52)	263	3.56 (9.85)	256	-2.6 (-4.03; -1.16)	-1.86 (-3.38; -0.35)	-0.64 (-4.51;3.23)
**Drugs Prescribed (Complex Multimorbidity Proxy)**							
< 10 drugs	1.14 (6.14)	253	2.29 (8.24)	230	-1.14 (-2.44; 0.15)	-1.15 (-2.52; 0.21)	
≥ 10 drugs	0.31 (9.38)	39	7.34 (14.66)	67	-7.04 (-12.22; -1.85)	-3.6 (-8.18; -0.98)	-3.75 (-6.9;-0.6)
**Depression**							
No	0.96 (6.99)	214	3.59 (10.49)	200	-2.63 (-4.34; -0.92)	-2.41 (-4.16; -0.66)	
Yes	1.23 (5.67)	78	3.09 (9.76)	97	-1.86 (-4.32; 0.6)	-0.86 (-2.91; 1.2)	1.81 (-0.79;4.4)
**Arrhythmias**							
No	0.89 (5.95)	257	3.2 (9.9)	259	-2.31 (-3.72; -0.9)	-1.87 (-3.38; -0.35)	
Yes	2.03 (10.54)	35	4.95 (12.39)	38	-2.92 (-8.31; 2.47)	-1.58 (-5.16; 2.01)	-0.28 (-3.8;3.23)
**Teaching practice**							
No	2.53 (10.87)	53	8.7 (15.04)	57	-6.17 (-11.17; -1.18)	-2.94 (-8.81; 2.93)	
Yes	0.7 (5.26)	239	2.18 (8.29)	240	-1.48 (-2.72; -0.23)	-1.5 (-2.84; -0.15)	1.95 (-1.99;5.88)
**Postgraduate medic trainer**							
No	1.79 (8.54)	91	4.6 (11.54)	121	-2.8 (-5.64; 0.3)	-2.09 (-5.22; 1.05)	
Yes	0.69 (5.58)	201	2.63 (9.2)	176	-1.94 (-3.46; -0.42)	-1.73 (-3.37; -0.1)	0.42 (-2.78;3.62)
