# Supplementary material for: How to Improve Healthcare for Patients with Multimorbidity and Polypharmacy in Primary Care: A Pragmatic Cluster-Randomized Clinical Trial of the MULTIPAP Intervention
Source: J Pers Med. 2022 May 6;12(5):752. doi: 10.3390/jpm12050752 (PMC9144280; doi:10.3390/jpm12050752)
Supplement: Supplementary file 1 [file jpm-12-00752-s001.zip › jpm-1629257-supplementary/Supplementary Table S6.pdf]

Supplementary Table S6. Secondary outcomes a 6 months.

	Control group	Intervention group	Adjusted difference in means	P value
	(N=295)	(N=298)		
**Use of health services** Median (RIQ)				
Hospital admissions	1 (1-2); n=287	1 (1-1); n=287	-0.098 (-0.644,0.449)	0.726
Emergency services	1 (1-2); n=287	1 (1-2); n=287	0.227 (-0.057,0.512)	0.118
Primary-care physician consultations	3 (2-6); n=287	4 (2-6); n=287	0.073 (-0.109,0.256)	0.432
Primary-care nurse consultations	2 (1-5); n=287	3 (2-6); n=287	0.168 (-0.081,0.416)	0.186
**Quality of life** mean(SD)				
EQ-5D-5 L (visual analogue scale)	68.5 (17.5); n=287	71.0 (18.4); n=285	2.21 (-0.72,5.13)	0.139
EQ-5D-5 L (index)	0.804 (0.170); n=287	0.777 (0.221); n=286	-0.016 (-0.041,0.010)	0.222
**Medication safety**				
Incidence of adverse drug reactions	1.0 (1-1); n=285	1.0 (1-1); n=287	0.49 (-0.30,1.28)	0.223
**Treatment adherence**				
Morisky-Green test	75/286 (26.2%)	75/287 (26.1%)	-0.19 (-0.69,0.32)	0.467
